# Capsaicin-Sensitive Sensory Nerves Are Necessary for the Protective Effect of Ghrelin in Cerulein-Induced Acute Pancreatitis in Rats

**DOI:** 10.3390/ijms18071402

**Published:** 2017-06-30

**Authors:** Joanna Bonior, Zygmunt Warzecha, Piotr Ceranowicz, Ryszard Gajdosz, Piotr Pierzchalski, Michalina Kot, Anna Leja-Szpak, Katarzyna Nawrot-Porąbka, Paweł Link-Lenczowski, Michał Pędziwiatr, Rafał Olszanecki, Krzysztof Bartuś, Rafał Trąbka, Beata Kuśnierz-Cabala, Artur Dembiński, Jolanta Jaworek

**Affiliations:** 1 Department of Medical Physiology, Faculty of Health Sciences, Jagiellonian University Medical College, 12 Michałowskiego St., 31-126 Krakow, Poland; joanna.bonior@uj.edu.pl (J.B.); piotr.pierzchalski@uj.edu.pl (P.P.); m.kot@uj.edu.pl (M.K.); a.leja-szpak@uj.edu.pl (A.L.-S.); k.nawrot-porabka@uj.edu.pl (K.N.-P.); p.link-lenczowski@uj.edu.pl (P.L.-L.); jolanta.jaworek@uj.edu.pl (J.J.); 2 Department of Physiology, Faculty of Medicine, Jagiellonian University Medical College, 16 Grzegórzecka St., 31-531 Krakow, Poland; mpwarzec@cyf-kr.edu.pl (Z.W.); mpdembin@cyf-kr.edu.pl (A.D.); 3 Department of Emergency Medical Care, Faculty of Health Sciences, Jagiellonian University Medical College, 12 Michałowskiego St., 31-126 Krakow, Poland; ryszard.gajdosz@uj.edu.pl; 4 2nd Department of Surgery, Faculty of Medicine, Jagiellonian University Medical College, 21 Kopernika St., 31-501 Krakow, Poland; michal.pedziwiatr@uj.edu.pl; 5 Department of Pharmacology, Faculty of Medicine, Jagiellonian University Medical College, 16 Grzegórzecka St., 31-531 Krakow, Poland; rafal.olszanecki@uj.edu.pl; 6 Department of Cardiovascular Surgery and Transplantology, Faculty of Medicine, Jagiellonian University, JP II Hospital, 80 Prądnicka St., 31-202 Krakow, Poland; krzysztof.bartus@uj.edu.pl; 7 Department of Rehabilitation, Faculty of Health Sciences, Jagiellonian University Medical College, 3 Koło Strzelnicy St., 30-219 Krakow, Poland; rafal.trabka@uj.edu.pl; 8 Department of Diagnostics, Chair of Clinical Biochemistry, Faculty of Medicine Jagiellonian University Medical College, 15 A Kopernika St., 31-501 Krakow, Poland; mbkusnie@cyf-kr.edu.pl

**Keywords:** sensory nerves, inflammation, acute pancreatitis, capsaicin, lipase, interleukin-4, tumor necrosis factor-α, heat shock protein 70

## Abstract

Ghrelin was shown to exhibit protective and therapeutic effect in the gut. Aim of the study was to investigate the role of sensory nerves (SN) in the protective effect of ghrelin in acute pancreatitis (AP). Studies were performed on male Wistar rats or isolated pancreatic acinar cells. After capsaicin deactivation of sensory nerves (CDSN) or treatment with saline, rats were pretreated intraperitoneally with ghrelin or saline. In those rats, AP was induced by cerulein or pancreases were used for isolation of pancreatic acinar cells. Pancreatic acinar cells were incubated in cerulein-free or cerulein containing solution. In rats with intact SN, pretreatment with ghrelin led to a reversal of the cerulein-induced increase in pancreatic weight, plasma activity of lipase and plasma concentration of tumor necrosis factor-α (TNF-α). These effects were associated with an increase in plasma interleukin-4 concentration and reduction in histological signs of pancreatic damage. CDSN tended to increase the severity of AP and abolished the protective effect of ghrelin. Exposure of pancreatic acinar cells to cerulein led to increase in cellular expression of mRNA for TNF-α and cellular synthesis of this cytokine. Pretreatment with ghrelin reduced this alteration, but this effect was only observed in acinar cells obtained from rats with intact SN. Moreover, CDSN inhibited the cerulein- and ghrelin-induced increase in gene expression and synthesis of heat shock protein 70 (HSP70) in those cells. Ghrelin exhibits the protective effect in cerulein-induced AP on the organ and pancreatic acinar cell level. Sensory nerves ablation abolishes this effect.

## 1. Introduction

Ghrelin is a 28-amino acid peptide originally isolated from the rat and human stomach and is formed from a 117-amino acid precursor [1,2,3]. Ghrelin acts via ghrelin receptor. Before the discovery of ghrelin this receptor was called growth hormone secretagogue receptor—GHS-R [1,3,4]. Early studies in humans and animals showed that ghrelin strongly stimulates the release of growth hormone from the pituitary gland [1]. This effect is a result of direct action of ghrelin on ghrelin receptors present on pituitary somatotrophs; however, ghrelin also stimulates the liberation of growth hormone indirectly by acting on growth hormone-releasing hormone (GH-RH) positive cells in the hypothalamus triggering GH-RH secretion [5]. In turn, GH-RH acting on the anterior part of the pituitary gland promotes the release of growth hormone. Another early discovered function of ghrelin is its effect on the energy balance of the body. Ghrelin induces body mass gain by increase in food intake and decrease in fat utilization [3,6,7].

The ghrelin receptor is present mainly in the pituitary gland and hypothalamus, but its existence was also found in other tissues of the body such as the thyroid gland, pancreas, spleen, myocardium, adrenal gland, gonad, heart, lung and cells of the immunological system [8,9,10]. Previous studies showed that ghrelin exhibits protective and therapeutic effect in the digestive tract. Pretreatment with this polypeptide inhibits the development of different experimental models of gastric lesions [11,12,13], as well as exhibits therapeutic effect in the course of oral [14], gastric [15,16] and duodenal [15,17] ulcers. In addition, most experimental studies on the role of ghrelin in colitis indicates that administration of this peptide exhibits protective and therapeutic effect in the large bowel [18,19,20,21,22,23]. Protective and therapeutic effect of ghrelin was also found in the pancreas. Pretreatment with ghrelin inhibits the development of acute pancreatitis induced by cerulein [24], taurocholate [25] and pancreatic ischemia followed by reperfusion [26]. Administration of ghrelin after induction of acute pancreatitis accelerates the recovery in this disease [27,28,29]. Moreover, treatment with this peptide attenuates the severity of acute lung injury in taurocholate-induced acute pancreatitis [30].

Primary unmyelinated capsaicin-sensitive sensory neurons play a nociceptive role and convey signals, mainly pain signals from the skin and internal environment of the body to the central nervous system. However, stimulation of these neurons also leads to release of neuromediators from their peripheral endings [31,32]. Capsaicin, a main pungent ingredient of chili pepper, binds to specific vanilloid (capsaicin) receptors present on capsaicin-sensitive sensory neurons [33,34]. This receptor is a nonselective cation channel and belongs to the transient receptor potential family. The current name of this receptor is the transient receptor potential vanilloid 1 (TRPV1) [33,34]. Low doses of capsaicin act on TRPV1 and stimulate primary sensory nerves by opening the nonselective cation channels. It leads to a local release of neuromediators such as calcitonin gene-related peptide (CGRP) and substance P [31,32,33]. In contrast, high neurotoxic doses of capsaicin lead to ablation of sensory nerves and decrease plasma and tissue level of CGRP [35]. Capsaicin-sensitive sensory nerves are involved in the maintenance of organ integrity in the gut. In the stomach, stimulation of sensory nerves by low doses of capsaicin or administration of exogenous CGRP before induction of ulcers exhibit a preventive effect in different experimental models of gastric ulcers [36,37], whereas the ablation of sensory nerves by neurotoxic doses of capsaicin aggravates gastric damage [38] and delays the healing of gastric ulcers [39].

Similar effects were observed in the pancreas. Ablation of capsaicin-sensitive sensory nerves before induction of acute pancreatitis increases the severity of this disease evoked by cerulein or pancreatic ischemia followed by reperfusion [40,41]. In contrast, stimulation of sensory nerves by low doses of capsaicin or pretreatment with CGRP before induction of acute pancreatitis exhibits protective effect in the pancreas and reduces the severity of cerulein- [42,43] and ischemia/reperfusion-induced acute pancreatitis [41]. On the other hand, administration of CGRP after induction of acute pancreatitis or persistent activity of sensory nerves increases the severity of acute pancreatitis and lead to functional insufficiency of the pancreas typical for chronic pancreatitis [44,45]. Moreover, there are studies showing that activation of TRPV1 may promote neurogenic inflammation in the pancreas via a release of substance P and activation of the neurokinin-1 receptor [46,47,48].

The aim of our present study was to investigate and compare the effect of intraperitoneal administration of ghrelin on the development of cerulein-induced acute pancreatitis in rats with intact sensory nerves or sensory nerve ablation evoked by neurotoxic doses of capsaicin. Moreover, this study was designated to assess the effect of ablation of sensory nerves and administration of ghrelin performed before isolation of pancreatic acinar cells on expression of mRNA for tumor necrosis factor-α (TNF-α) and heat shock protein 70 (HSP70), and synthesis of those proteins by pancreatic acinar cells incubated in the cerulein-containing or cerulein-free medium.

## 2. Results

### 2.1. In Vivo Studies

Subcutaneous infusion of cerulein at a dose of 5 μg/kg/h for 5 h (1 mL/h), caused acute edematous pancreatitis in all animals subjected to this procedure.

#### 2.1.1. Effect of Ghrelin Administered Intraperitoneally on Pancreatic Weight and Histological Signs of Acute Pancreatitis

**Figure 1:**
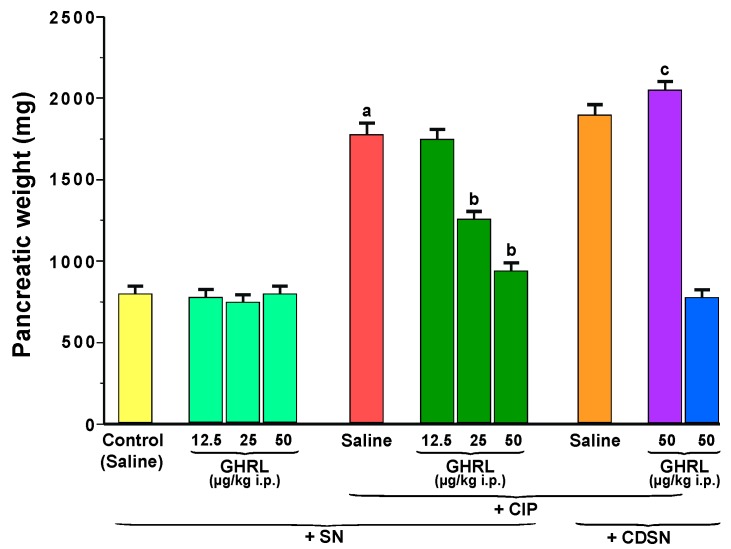
Effect of saline or ghrelin (GHRL) given intraperitoneally at a dose of 12.5, 25 or 50 µg/kg and cerulein-induced pancreatitis (CIP) on pancreatic weight in sensory nerves-intact rats (SN) or rats with capsaicin deactivation of sensory nerves (CDSN). ^a^ *p* < 0.05 compared to the control group with intact sensory nerves and without induction of CIP; ^b^ *p* < 0.05 compared to sensory nerves-intact rats with CIP pretreated with saline; ^c^ *p* < 0.05 compared to sensory nerves-intact rats pretreated with GHRL at a dose of 50 µg/kg prior to CIP. Mean ± standard error of the mean (SEM) from values obtained from 10–15 rats in each experimental group.

In rats from the control sensory nerves-intact group treated with saline, pancreatic weight reached 800 ± 65 mg (Figure 1). Intraperitoneal administration of increasing doses of ghrelin, 12.5, 25 or 50 μg/kg, failed to affect significantly this parameter in sensory nerves-intact rats without induction of acute pancreatitis.

Subcutaneous infusion of cerulein induced pancreatic edema and significantly increased pancreatic weight in sensory nerves-intact rats to a level of 1780 ± 150 mg. Intraperitoneal administration of ghrelin at a doses of 25 or 50 µg/kg, 30 min prior to the commencement of cerulein infusion, resulted in a statistically significant reduction in the cerulein-induced increase in pancreatic weight in rats with intact sensory nerves, to a level of 1259 ± 95 mg and 944 ± 65 mg, respectively (Figure 1). Ghrelin given at a dose of 12.5 µg/kg was without a significant effect on pancreatic weight in sensory nerves-intact rats infused subcutaneously with cerulein.

Capsaicin deactivation of sensory nerves (CDSN) tended to enhance the cerulein-evoked increase in pancreatic edema and pancreatic weight. However, the difference between pancreatic weight in sensory nerves-intact rats treated with saline before cerulein infusion and rats with CDSN treated with saline before cerulein infusion was not statistically significant.

CDSN abolished the ghrelin-induced reduction in cerulein-evoked increase in pancreatic weight. In rats with CDSN pretreated with ghrelin prior to the development of cerulein-induced pancreatitis (CIP), pancreatic weight reached a value of 2050 ± 150 mg and was even slightly increased in comparison to pancreatic weight observed in sensory nerves-intact rats without pretreatment with ghrelin before induction of acute pancreatitis or rats with CDSN pretreated with saline before CIP. Pancreatic weight in rats with CDSN treated with ghrelin given at a dose of 50 µg/kg before induction of CIP was significantly higher than that observed in sensory nerves-intact rats pretreated with the same dose of ghrelin before CIP development.

Administration of ghrelin at a dose of 50 µg/kg did not alter pancreatic weight in rats with CDSN without CIP (Figure 1).

**Figure 2:**
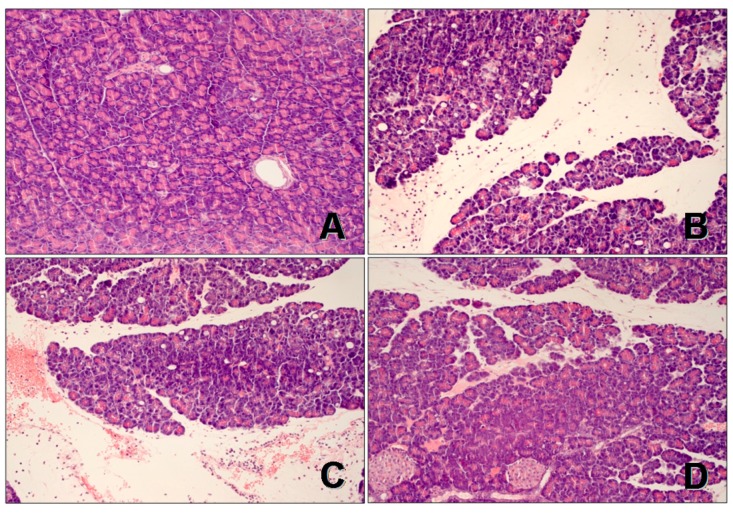
Histological images of pancreatic tissues stained with hematoxylin and eosin, magnification 400×: (**A**) control sensory nerves-intact rats treated with saline without cerulein-induced pancreatitis (CIP); (**B**) sensory nerves-intact rats treated with saline followed by CIP development; (**C**) rats with capsaicin deactivation of sensory nerves treated with saline followed by CIP development; and (**D**) Sensory nerves-intact rats treated with ghrelin given at a dose of 50 µg/kg followed by CIP development.

**Figure 3:**
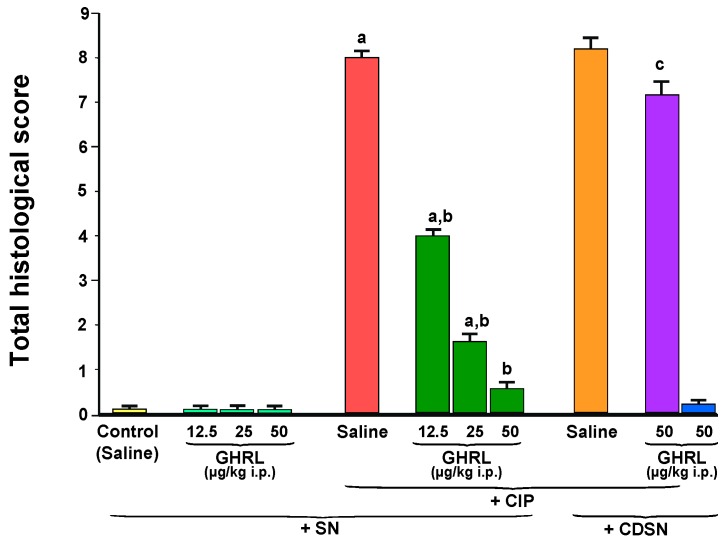
Effect of saline or ghrelin (GHRL) given intraperitoneally at a dose of 12.5, 25 or 50 µg/kg and cerulein-induced pancreatitis (CIP) on total histological score in sensory nerves-intact rats (SN) or rats with capsaicin deactivation of sensory nerves (CDSN). ^a^ *p* < 0.05 compared to the control group with intact sensory nerves and without induction of CIP; ^b^ *p* < 0.05 compared to sensory nerves-intact rats with CIP pretreated with saline; ^c^ *p* < 0.05 compared to sensory nerves-intact rats pretreated with GHRL at a dose of 50 µg/kg prior to CIP. Mean ± SEM from values obtained from 10–15 rats in each experimental group.

Pancreases, obtained from control sensory nerves-intact rats treated with saline without CIP, were characterized by normal morphology in both, macro- and microscopic evaluation (Figure 2 and Figure 3, Table 1). In sensory nerves-intact rats without induction of CIP, administration of ghrelin at doses used failed to affect morphology of the pancreas (Figure 3, Table 1).

In sensory nerves-intact rats pretreated with saline, infusion with cerulein led to the development of acute pancreatitis. Macroscopically it was manifested as a marked swelling of the pancreas. Microscopic evaluation revealed pancreatic interlobular edema and moderate or severe intralobular edema accompanied by moderate perivascular and scarce diffuse inflammatory infiltration or abundant diffuse inflammatory infiltration. In most cases, vacuolization was observed in more than 50% of acinar cells. No necrosis or hemorrhage was observed. In these groups of rats, total histological score reached a value of 7.60 ± 0.27 (Figure 3). In rats with intact sensory nerves, administration of ghrelin before generation of CIP led to reduction in morphological signs of acute pancreatitis, as compared to sensory nerves-intact rats treated with saline before induction of CIP (Figure 2 and Figure 3, Table 1).

CDSN performed prior to induction of CIP tended to increase the severity of acute pancreatitis in comparison to findings observed in sensory nerves-intact rats with development of CIP, but this effect was statistically insignificant (Figure 2 and Figure 3, Table 1).

The usage of ghrelin, at a selected dose of 50 µg/kg, prior to the start of cerulein infusion in the group of animals with CDSN, failed to improve the morphology of the pancreas. Morphological signs of pancreatic damage in this group of animals were significantly bigger than that observed in sensory nerves-intact rats treated with ghrelin at a dose of 50 µg/kg before induction of CIP.

The use of ghrelin without induction of CIP did not affected pancreatic morphology in rats with intact sensory nerves or rats with CDSN (Figure 3, Table 1).

#### 2.1.2. Plasma Lipase Activity

**Figure 4:**
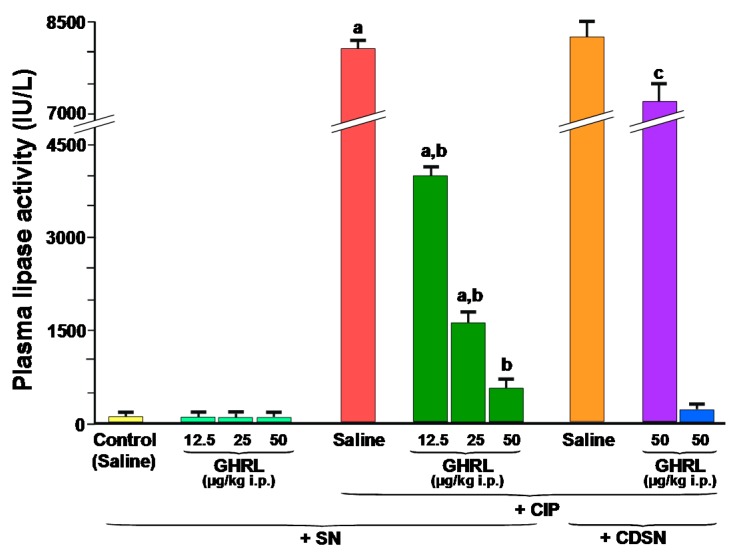
Effect of saline or ghrelin (GHRL) given intraperitoneally at a dose of 12.5, 25 or 50 µg/kg and cerulein-induced pancreatitis (CIP) on plasma activity of lipase in sensory nerves-intact rats (SN) or rats with capsaicin deactivation of sensory nerves (CDSN). ^a^ *p* < 0.05 compared to the control group with intact sensory nerves and without induction of CIP ; ^b^ *p* < 0.05 compared to sensory nerves-intact rats with CIP pretreated with saline; ^c^ *p* < 0.05 compared to sensory nerves-intact rats pretreated with GHRL at a dose of 50 µg/kg prior to CIP. Mean ± SEM from values obtained from 10–15 rats in each experimental group.

Plasma lipase activity in control sensory nerves-intact rats treated with saline without induction of CIP was 100 ± 15 IU/L and remained unchanged following administration of increasing doses of ghrelin, 12.5, 25 or 50 µg/kg (Figure 4).

In sensory nerves-intact rats, induction of acute pancreatitis by cerulein caused a dramatic, statistically significant increase in plasma activity of lipase to around 8000 IU/L. In rats with intact sensory nerves, administration of ghrelin at increasing doses, 12.5, 25 or 50 µg/kg before the induction of CIP, resulted in a statistically significant decrease in plasma activity of this pancreatic digestive enzyme to approximately 4000, 1600 and 650 IU/L, respectively (Figure 4).

CDSN tended to increase plasma activity of lipase in rats with CIP, but this effect was statistically insignificant. Administration of ghrelin at a dose of 50 µg/kg before the start of cerulein infusion was without significant effect on plasma activity of lipase in rats with CDSN and CIP.

In addition, administration of ghrelin did not affect plasma activity of lipase in rats with CDSN, but without induction of CIP (Figure 4).

#### 2.1.3. Pancreatic Blood Flow

**Figure 5:**
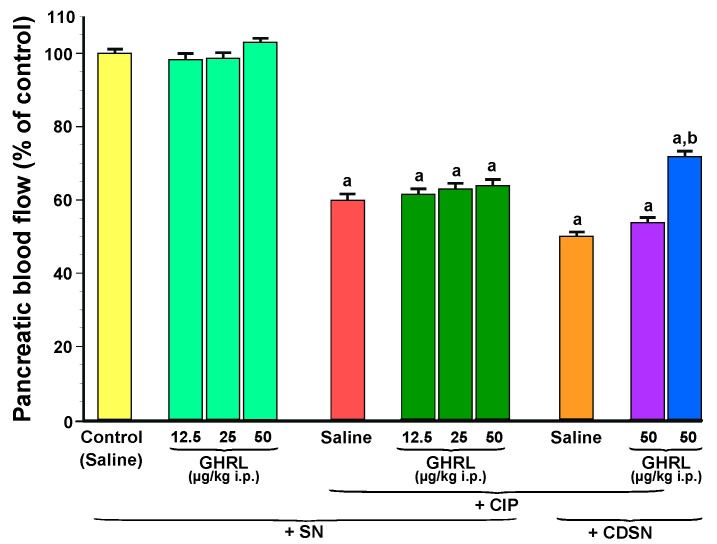
Effect of saline or ghrelin (GHRL) given intraperitoneally at a dose of 12.5, 25 or 50 µg/kg and cerulein-induced pancreatitis (CIP) on pancreatic blood flow in sensory nerves-intact rats (SN) or rats with capsaicin deactivation of sensory nerves (CDSN). ^a^ *p* < 0.05 compared to the control group with intact sensory nerves and without induction of CIP; ^b^ *p* < 0.05 compared to sensory nerves-intact rats pretreated with GHRL at a dose of 50 µg/kg without induction of CIP. Mean ± SEM from values obtained from 10–15 rats in each experimental group.

Intraperitoneal administration of increasing doses of ghrelin, 12.5, 25 or 50 µg/kg, did not affect pancreatic blood flow in sensory nerves-intact rats without induction of acute pancreatitis (Figure 5).

In sensory nerves-intact rats, the development of CIP significantly reduced pancreatic blood flow by about 40%. Pretreatment with ghrelin at doses used, 30 min before the start of cerulein infusion, failed to significantly affect pancreatic blood flow in sensory nerves-intact rats or rats with CDSN. CDSN in rats pretreated with saline before CIP tended to further decrease in pancreatic blood flow, but this effect was statistically insignificant. On the other hand, we found a significant difference in pancreatic blood flow between two groups of rats without CIP: rats with intact sensory nerves treated with ghrelin given at a dose of 50 µg/kg and rats with CDSN treated with the same dose of this polypeptide. CDSN reduced pancreatic blood flow by almost 30% (Figure 5).

#### 2.1.4. Plasma Concentration of Tumor Necrosis Factor-α (TNF-α) and Interleukin-4

**Figure 6:**
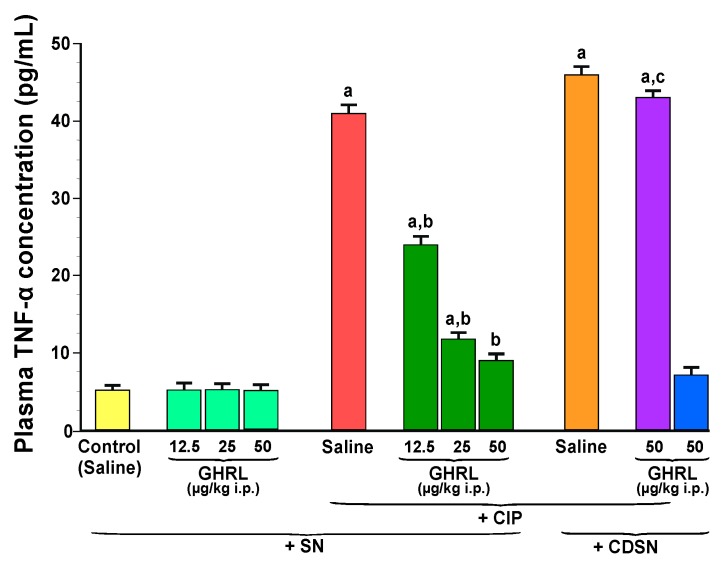
Effect of saline or ghrelin (GHRL) given intraperitoneally at a dose of 12.5, 25 or 50 µg/kg and cerulein-induced pancreatitis (CIP) on plasma concentration of tumor necrosis factor-α (TNF-α) in sensory nerves-intact rats (SN) or rats with capsaicin deactivation of sensory nerves (CDSN). ^a^ *p* < 0.05 compared to the control group with intact sensory nerves and without induction of CIP; ^b^ *p* < 0.05 compared to sensory nerves-intact rats with CIP pretreated with saline; ^c^ *p* < 0.05 compared to sensory nerves-intact rats pretreated with GHRL at a dose of 50 µg/kg prior to CIP. Mean ± SEM from values obtained from 10–15 rats in each experimental group.

Plasma concentration of the pro-inflammatory cytokine, TNF-α in the control group of animals with intact sensory nerves and treated with saline was 5.1 ± 0.5 pg/mL and remained unchanged following treatment with increasing doses of ghrelin, 12.5, 25 or 50 µg/kg (Figure 6).

CIP resulted in a statistically significant increase in the concentration of TNF-α up to 41.0 ± 7.0 pg/mL. Increasing doses of ghrelin, 12.5, 25 or 50 µg/kg given intraperitoneally 30 min prior to the induction of CIP, resulted in a statistically significant reduction in plasma concentration of TNF-α to the level of 24.0 ± 2.5, 11.8 ± 1.5, and 9.0 ± 1.0 pg/mL, respectively (Figure 6).

In rats with CDSN treated with saline before induction of CIP, plasma concentration of TNF-α reached a value of 46.0 ± 8.0 pg/mL. This concentration of TNF-α was higher than that observed in sensory nerves-intact rats treated with saline before induction of CIP, but this difference was statistically insignificant. In rats with CDSN, administration of ghrelin at a dose of 50 µg/kg prior to start of cerulein infusion did not significantly affect plasma level of TNF-α. For this reason, there was a statistically significant difference in plasma level of TNF-α between sensory nerves-intact rats pretreated with ghrelin at a dose of 50 µg/kg before induction of CIP and rats with CDSN pretreated with the same dose of ghrelin before induction of CIP. Pretreatment with ghrelin was without effect on plasma concentration of TNF-α in rats with CDSN without CIP (Figure 6).

**Figure 7:**
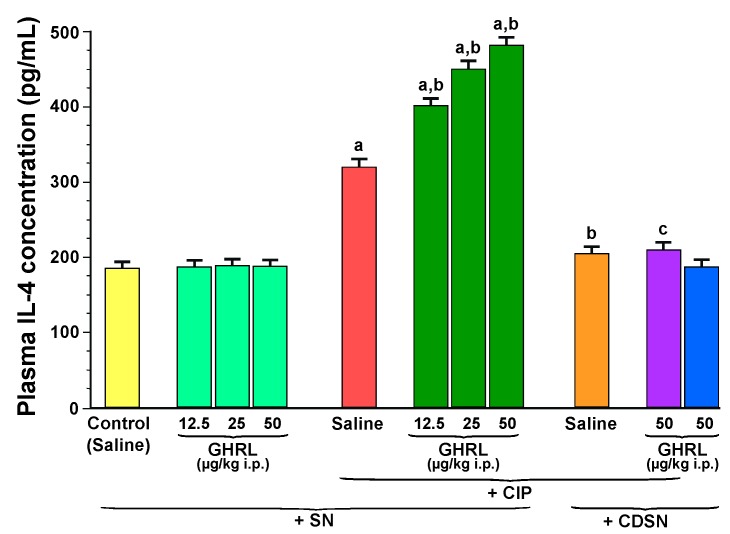
Effect of saline or ghrelin (GHRL) given intraperitoneally at a dose of 12.5, 25 or 50 µg/kg and cerulein-induced pancreatitis (CIP) on plasma concentration of interleukin-4 (IL-4) in sensory nerves-intact rats (SN) or rats with capsaicin deactivation of sensory nerves (CDSN). ^a^ *p* < 0.05 compared to the control group with intact sensory nerves and without induction of CIP; ^b^ *p* < 0.05 compared to sensory nerves-intact rats with CIP pretreated with saline; ^c^ *p* < 0.05 compared to sensory nerves-intact rats pretreated with GHRL at a dose of 50 µg/kg prior to CIP. Mean ± SEM from values obtained from 10–15 rats in each experimental group.

Plasma concentration of anti-inflammatory cytokine, interleukin-4 in control, sensory nerves-intact rats treated with saline without CIP was 185 ± 20 pg/mL and remained unchanged after administration of ghrelin at doses used (Figure 7).

CIP resulted in a statistically significant increase in the concentration of interleukin-4 to 320 ± 25 pg/mL in rats with intact sensory nerves. Administration of ghrelin at a dose of 12.5, 25 or 50 µg/kg before the induction of CIP, led to an additional and statistically significant increase in plasma interleukin-4 concentration to a level of 410 ± 50, 450 ± 55 and 482 ± 60 pg/mL, respectively (Figure 7).

In contrast to effects observed in sensory nerves-intact rats, capsaicin deactivation of sensory nerves abolished the CIP-evoked increase in plasma level of interleukin-4 in rats pretreated with saline, as well as the stimulatory effect on the release of this anti-inflammatory cytokine evoked by the combination of ghrelin plus CIP. Administration of ghrelin was also without any effect on plasma concentration of interleukin-4 in rats with CDSN without induction of acute pancreatitis (Figure 7).

### 2.2. In Vitro Studies Performed on Isolated Pancreatic Acinar Cells Obtained from Rats with Intact or Deactivated Sensory Nerves

#### 2.2.1. Determination of *TNF-α* Gene Expression and Protein Synthesis

**Figure 8:**
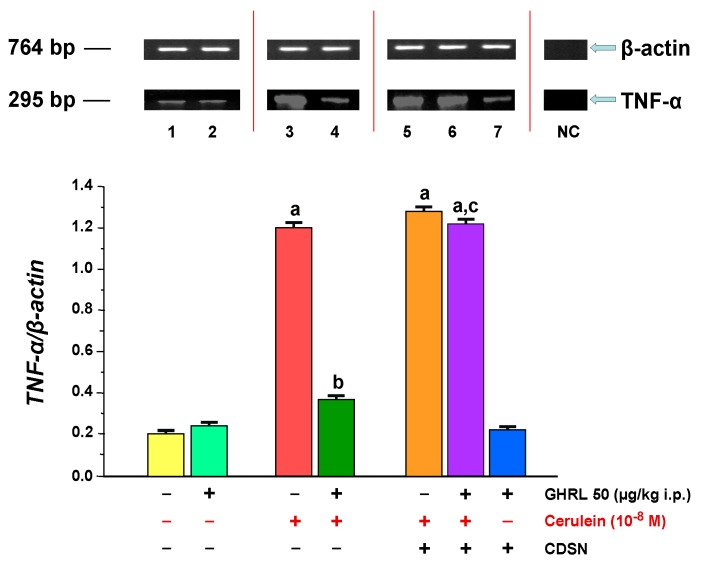
Analysis of *tumor necrosis factor-α* (*TNF-α*) gene expression assessed using reverse transcription-polymerase chain reaction (RT-PCR) and densitometric analysis of TNF-α/β-actin mRNA ratio in isolated pancreatic acinar cells: (lane 1) acinar cells obtained from control sensory nerves (SN)-intact rats treated with saline, after isolation, acinar cells incubated in cerulein-free solution; (lane 2) acinar cells obtained from SN-intact rats treated with ghrelin (GHRL), after isolation, acinar cells incubated in cerulein-free solution; (lane 3) acinar cells obtained from SN-intact rats treated with saline, after isolation, acinar cells incubated in solution containing cerulein at a concentration of 10^−8^ M; (lane 4) acinar cells obtained from SN-intact rats treated with GHRL, after isolation, acinar cells incubated in solution containing cerulein at a concentration of 10^−8^ M; (lane 5) acinar cells obtained from rats with capsaicin deactivation of SN (CDSN) and treated with saline, after isolation, acinar cells incubated in solution containing cerulein at a concentration of 10^−8^ M; (lane 6) acinar cells obtained from rats with CDSN and treated with GHRL, after isolation, acinar cells incubated in solution containing cerulein at a concentration of 10^−8^ M; (lane 7) acinar cells obtained from rats with CDSN and treated with GHRL, after isolation, acinar cells incubated in cerulein-free solution. NC = negative control. Reference gene: *β-actin*. ^a^ *p* < 0.05 compared to control acinar cells obtained from rats with intact SN (lane 1); ^b^ *p* < 0.05 compared to acinar cells stimulated with cerulein after isolation from SN-intact rats treated with saline (line 3); ^c^ *p* < 0.05 compared to acinar cells stimulated with cerulein after isolation from SN-intact rats treated with GHRL (lane 4). In each experimental group, there was at least six observations.

The gene expression of pro-inflammatory cytokine, *TNF-α* in isolated rat pancreatic acinar cells in the in vitro model was detected in all samples tested. In acinar cells, obtained from control sensory nerves-intact rats treated with saline and after isolation incubated in cerulein-free solution, the ratio of TNF-α/β-actin mRNA signal was 0.20 ± 0.02 (Figure 8). Intraperitoneal administration of ghrelin before isolation of acinar cells from rats with intact sensory nerves did not significantly alter the ratio of *TNF-α/β-actin* gene expression in comparison to control group (0.24 ± 0.02 versus 0.20 ± 0.02).

Hyperstimulation of pancreatic acinar cells with cerulein given at a concentration of 10^−8^ M resulted in a statistically significant increase in *TNF-α* gene expression, the ratio of mRNA for TNF-α to mRNA for β-actin reached a level of 1.20 ± 0.06. Peripheral administration of ghrelin, before isolation of acinar cells from sensory intact rats, and incubation of those acinar cells with cerulein resulted in a statistically significant decrease in expression of mRNA for TNF-α. The ratio of TNF-α/β-actin mRNA was 0.37 ± 0.03 (Figure 8).

CDSN performed before treatment with ghrelin, abolished the inhibitory effect of ghrelin on *TNF-α* gene expression in isolated acinar cells stimulated with cerulein. Comparison of gene expression for *TNF-α* in pancreatic acinar cells obtained from rats with intact sensory nerves treated with ghrelin and rats with CDSN treated with ghrelin showed no difference between them (Figure 8).

**Figure 9:**
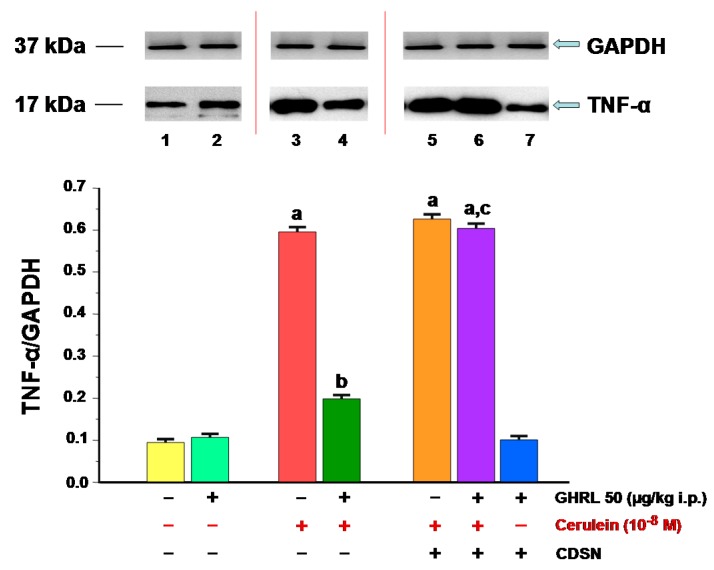
Analysis of tumor necrosis factor-α (TNF-α) protein synthesis determined by the method of immunoblotting and densitometric analysis of TNF-α/glyceraldehyde-3-phosphate dehydrogenase (GAPDH) protein ratio in isolated pancreatic acinar cells: (lane 1) acinar cells obtained from control sensory nerves (SN)-intact rats treated with saline, after isolation, acinar cells incubated in cerulein-free solution; (lane 2) acinar cells obtained from SN-intact rats treated with ghrelin (GHRL), after isolation, acinar cells incubated in cerulein-free solution; (lane 3) acinar cells obtained from SN-intact rats treated with saline, after isolation, acinar cells incubated in solution containing cerulein at a concentration of 10^−8^ M; (lane 4) acinar cells obtained from SN-intact rats treated with GHRL, after isolation, acinar cells incubated in solution containing cerulein at a concentration of 10^−8^ M; (lane 5) acinar cells obtained from rats with capsaicin deactivation of SN (CDSN) and treated with saline, after isolation, acinar cells incubated in solution containing cerulein at a concentration of 10^−8^ M; (lane 6) acinar cells obtained from rats with CDSN and treated with GHRL, after isolation, acinar cells incubated in solution containing cerulein at a concentration of 10^−8^ M; (lane 7) acinar cells obtained from rats with CDSN and treated with GHRL, after isolation, acinar cells incubated in cerulein-free solution. ^a^ *p* < 0.05 compared to control acinar cells obtained from rats with intact SN (lane 1); ^b^ *p* < 0.05 compared to acinar cells stimulated with cerulein after isolation from SN-intact rats treated with saline (line 3); ^c^ *p* < 0.05 compared to acinar cells stimulated with cerulein after isolation from SN-intact rats treated with GHRL (lane 4). In each experimental group, there was at least six observations.

The presence of the pro-inflammatory cytokine, TNF-α protein has been demonstrated in isolated pancreatic acinar cells in the in vitro model in all animal groups (Figure 9). The ratio value calculated for TNF-α/GAPDH (Glyceraldehyde-3-Phosphate Dehydrogenase) protein synthesis in isolated acinar cells incubated in cerulein-free solution and obtained from control sensory nerves-intact rats treated with saline was 0.098 ± 0.004. Peripheral administration of ghrelin to rats with intact sensory nerves prior to the in vitro experiment, did not significantly affect the ratio of TNF-α/GAPDH protein, which remained at the level of 0.11 ± 0.01 (Figure 9).

The stimulation of isolated pancreatic acinar cells with cerulein, given at a supramaximal, previously selected concentration of 10^−8^ M for 5 h, resulted in a statistically significant in increase synthesis of TNF-α protein. This effect reached a similar value in acinar cells obtained from sensory nerves-intact rats treated with saline and rats with CDSN and treated with saline. The ratio of TNF-α protein/GAPDH protein was in those cells 0.59 ± 0.05 and 0.63 ± 0.06, respectively (Figure 9).

Intraperitoneal administration of ghrelin, before isolation of pancreatic acinar cells, in rats with intact sensory nerves significantly reversed the cerulein-induced increase in synthesis of TNF-α in those cells. The ratio of TNF-α protein to GAPDH protein reached a value 0.20 ± 0.02 (Figure 9). In contrast, the cerulein-induced increase in TNF-α synthesis was not affected by pretreatment with ghrelin in acinar cells obtained from rats with CDSN.

CDSN failed to affect synthesis of TNF-α in acinar cells incubated in cerulein-free medium after isolation from rats pretreated with ghrelin. The ratio TNF-α/GAPDH in those cells reached a value of 0.10 ± 0.01 and was similar to that observed in acinar cells isolated from sensory nerves-intact rats pretreated with ghrelin and after isolation, incubated in cerulein-free medium (Figure 9).

#### 2.2.2. Determination of *Heat Shock Protein 70 (HSP70)* Gene Expression and Protein Synthesis

**Figure 10:**
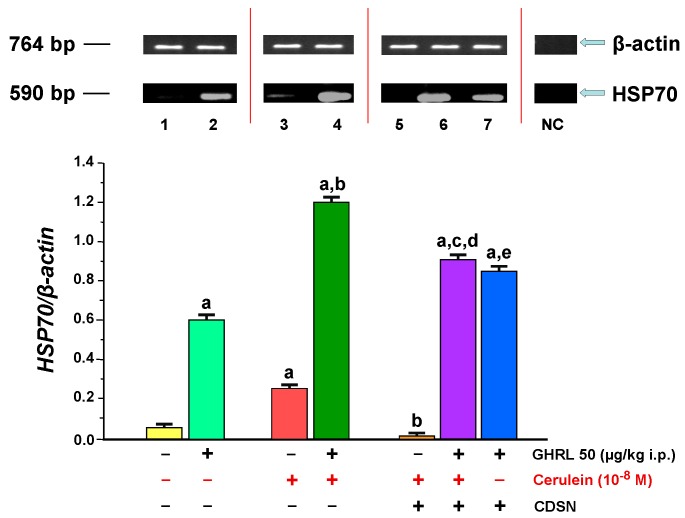
Analysis of *heat shock protein 70 (HSP70)* gene expression assessed using RT-PCR and densitometric analysis of HSP70/β-actin mRNA ratio in isolated pancreatic acinar cells: (lane 1) acinar cells obtained from control sensory nerves (SN)-intact rats treated with saline, after isolation, acinar cells incubated in cerulein-free solution; (lane 2) acinar cells obtained from SN-intact rats treated with ghrelin (GHRL), after isolation, acinar cells incubated in cerulein-free solution; (lane 3) acinar cells obtained from SN-intact rats treated with saline, after isolation, acinar cells incubated in solution containing cerulein at a concentration of 10^−8^ M; (lane 4) acinar cells obtained from SN-intact rats treated with GHRL, after isolation, acinar cells incubated in solution containing cerulein at a concentration of 10^−8^ M; (lane 5) acinar cells obtained from rats with capsaicin deactivation of SN (CDSN) and treated with saline, after isolation, acinar cells incubated in solution containing cerulein at a concentration of 10^−8^ M; (lane 6) acinar cells obtained from rats with CDSN and treated with GHRL, after isolation, acinar cells incubated in solution containing cerulein at a concentration of 10^−8^ M; (lane 7) acinar cells obtained from rats with CDSN and treated with GHRL, after isolation, acinar cells incubated in cerulein-free solution. NC = negative control. Reference gene: *β-actin*. ^a^ *p* < 0.05 compared to control acinar cells obtained from rats with intact SN (lane 1); ^b^ *p* < 0.05 compared to acinar cells stimulated with cerulein after isolation from SN-intact rats treated with saline (line 3); ^c^ *p* < 0.05 compared to acinar cells stimulated with cerulein after isolation from rats with CDSN and treated with saline (lane 5); ^d^ *p* < 0.05 compared to acinar cells stimulated with cerulein after isolation from SN-intact rats treated with GHRL (lane 4); ^e^ *p* < 0.05 compared to acinar cells incubated in cerulein-free solution after isolation from SN-intact rats treated with GHRL (lane 2). In each experimental group, there was at least six observations.

The presence of HSP70 mRNA signal was demonstrated in vitro in isolated pancreatic acinar cells obtained from all experimental groups. In pancreatic cells obtained from sensory nerves-intact rats pretreated with saline (control group), the ratio of *HSP70/β-actin* gene expression was 0.05 ± 0.001. Peritoneal application of ghrelin to rats 48 h prior to in vitro experiment, led to a statistically significant increase the ratio of *HSP70/β-actin* gene expression to the level of 0.60 ± 0.03 (Figure 10).

Stimulation of rat pancreatic acinar cells with a selected supramaximal concentration of cerulein, 10^−8^ M for 5 h, resulted in a statistically significant increase in the ratio HSP70/β-actin mRNA to the level of 0.25 ± 0.01. In the in vivo model, intraperitoneal administration of ghrelin to rats, at a dose of 50 μg/kg, 48 h before the use of the pancreatic secretagogue in vitro, resulted in a statistically significant increase in a value ratio of HSP70/β-actin mRNA to 1.20 ± 0.06 (Figure 10).

CDSN before isolation of acinar cells from rats treated with saline produced a significant decrease in the ratio of HSP70/β-actin mRNA in pancreatic acinar cells stimulated with cerulein in comparison to a value observed in acinar cells obtained from sensory nerves-intact rats treated with saline (0.01 ± 0.001 versus 1.20 ± 0.06).

Intraperitoneal administration of ghrelin to animals with CDSN in vivo prior to the administration of cerulein at a concentration of 10^−8^ M in vitro, resulted in a statistically significant increase in the ratio values of HSP70/β-actin mRNA signal in comparison to the group without administration of ghrelin. However, this ratio was still significantly lower than that observed in acinar cells incubated with cerulein after isolation from sensory intact-rats treated with ghrelin (0.91 ± 0.04 versus 1.20 ± 0.06). On the other hand, the ratio HSP70/β-actin mRNA is significantly lower in acinar cells incubated in cerulein-free solution after isolation from sensory nerves-intact rats treated with ghrelin than that observed in acinar cells incubated in cerulein-free solution after isolation from rats with CDSN and treated with ghrelin (0.60 ± 0.03 versus 0.85 ± 0.04) (Figure 10).

**Figure 11:**
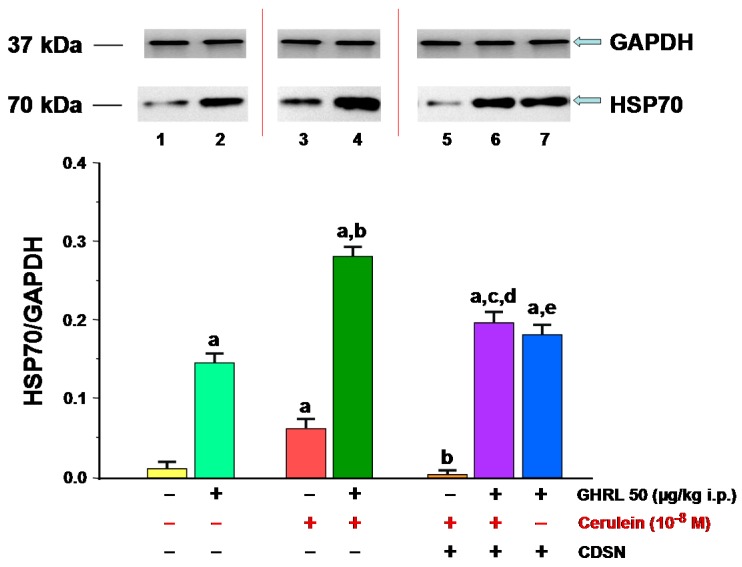
Analysis of the HSP70 protein synthesis determined by the method of immunoblotting and densitometric analysis of TNF-α/GAPDH protein ratio in isolated pancreatic acinar cells : (lane 1) acinar cells obtained from control sensory nerves (SN)-intact rats treated with saline, after isolation, acinar cells incubated in cerulein-free solution; (lane 2) acinar cells obtained from SN-intact rats treated with ghrelin (GHRL), after isolation, acinar cells incubated in cerulein-free solution; (lane 3) acinar cells obtained from SN-intact rats treated with saline, after isolation, acinar cells incubated in solution containing cerulein at a concentration of 10^−8^ M; (lane 4) acinar cells obtained from SN-intact rats treated with GHRL, after isolation, acinar cells incubated in solution containing cerulein at a concentration of 10^−8^ M; (lane 5) acinar cells obtained from rats with capsaicin deactivation of SN (CDSN) and treated with saline, after isolation, acinar cells incubated in solution containing cerulein at a concentration of 10^−8^ M; (lane 6) acinar cells obtained from rats with CDSN and treated with GHRL, after isolation, acinar cells incubated in solution containing cerulein at a concentration of 10^−8^ M; (lane 7) acinar cells obtained from rats with CDSN and treated with GHRL, after isolation, acinar cells incubated in cerulein-free solution. ^a^ *p* < 0.05 compared to control acinar cells obtained from rats with intact SN (lane 1); ^b^ *p* < 0.05 compared to acinar cells stimulated with cerulein after isolation from SN-intact rats treated with saline (line 3); ^c^ *p* < 0.05 compared to acinar cells stimulated with cerulein after isolation from rats with CDSN and treated with saline (lane 5); ^d^ *p* < 0.05 compared to acinar cells stimulated with cerulein after isolation from SN-intact rats treated with GHRL (lane 4); ^e^ *p* < 0.05 compared to acinar cells incubated in cerulein-free solution after isolation from SN-intact rats treated with GHRL (lane 2). In each experimental group, there was at least six observations.

In the rat isolated pancreatic acinar cells in the in vitro model, the presence of HSP70 was detected in all studied groups (Figure 11). The ratio of HSP70/GAPDH protein in the control group (acinar cells obtained from sensory nerves-intact rats treated with saline, after isolation, acinar cells incubated in cerulein-free solution) was 0.01 ± 0.001. Peripheral administration of ghrelin to sensory nerves-intact rats prior to in vitro experiment, resulted in a statistically significant increase the ratio value to 0.15 ± 0.01.

Stimulation of pancreatic acinar cells via pancreatic secretagogue, cerulein given at a concentration of 10^−8^ M for 5 h led to a statistically significant increase in cellular synthesis of HSP70 to a level of 0.06 ± 0.003 compared to HSP70 production observed in control acinar cells incubated in cerulein-free solution. In vivo administration of ghrelin prior to cerulein usage in vitro resulted in a statistically significant increase in the ratio of HSP70/GAPDH protein to a value of 0.28 ± 0.02 (Figure 11).

CDSN caused a dramatic, statistically significant decrease in ratio of HSP70 to GAPDH in the pancreatic acinar cells stimulated by cerulein, at a concentration of 10^−8^ M, to the level of 0.003 ± 0.0001, as compared to acinar cells incubated in cerulein containing solution and obtained from sensory nerves-intact rats. In rats with CDSN, intraperitoneal administration of ghrelin in vivo at a dose of 50.0 μg/kg, 48 h prior to the in vitro incubation in cerulein containing solution (10^−8^ M), resulted in a statistically significant increase of the ratio of HSP70 protein to GAPDH protein to the value of 0.20 ± 0.02 versus the groups without treatment with ghrelin. However, it still remained significantly smaller compared to ratio observed in acinar cells obtained from sensory nerves-intact animals receiving ghrelin at the same dose, followed by incubation of acinar cells in cerulein containing solution (0.28 ± 0.02). On the other hand, the ratio of HSP70/GAPDH protein signals in pancreatic acinar cells incubated in cerulein-free solution after isolation from animals with CDSN and treated with ghrelin was significantly higher than that ratio observed in pancreatic acinar cells incubated in cerulein-free solution after isolation from rats with intact sensory nerves and treated with ghrelin (0.18 ± 0.01 versus 0.15 ± 0.01) (Figure 11).

## 3. Discussion

Previous studies showed that pretreatment with ghrelin inhibits the development of acute pancreatitis evoked by cerulein, taurocholate or pancreatic ischemia followed by organ reperfusion [24,25,26]. Our current study confirms and extends these observations. Administration of cerulein led to the development of acute edematous pancreatitis. Pancreases were swollen, mean pancreatic weight was increased by more than 120%. Microscopic examination showed interlobular and moderate or severe intralobular pancreatic edema. These alterations were accompanied by perivascular and diffuse inflammatory infiltration, and vacuolization of acinar cells. Pretreatment with ghrelin performed in rats with intact capsaicin-sensitive sensory nerves reduced the severity of cerulein-induced acute pancreatitis. It reduced pancreatic edema and other histological signs of pancreatitis. Reduction in pancreatic edema was also found as a decrease in pancreatic weight. Moreover, pretreatment with ghrelin reduced the pancreatitis-evoked increase in plasma activity of pancreatic digestive enzyme, lipase, and plasma concentration of pro-inflammatory cytokine, tumor necrosis factor-α, whereas plasma concentration of anti-inflammatory cytokine, interleukin-4 was enhanced.

The increase in plasma activity of lipase is a well-known index of acute pancreatitis development and the severity of this disease with high sensitivity and specificity [49,50]. In our present study, induction of acute pancreatitis led to eighty-fold increase in plasma activity of lipase in comparison to the control value. Pretreatment with ghrelin decreased the pancreatitis-evoked increase in plasma activity of that pancreatic digestive enzyme. This effect seems to be a result as well as a mechanism of protective effect of ghrelin in the pancreas, which indicates that ghrelin prevents entering pancreatic enzymes to interstitial space of the pancreas and circulation. Moreover, it was found that pancreatic digestive enzymes, especially proteases induce leukocyte-endothelial adhesion leading to inflammatory infiltration and microcirculatory failure in the pancreas [51]. In acute pancreatitis, leukocytes adhere to endothelium, infiltrate pancreatic tissue and produce pro-inflammatory cytokines within this organ [52,53]. These data are in agreement with our present observations. In rats with intact sensory nerves, pretreatment with ghrelin reduced the cerulein-evoked increase in plasma level of lipase and this effect was associated with reduction of leukocyte infiltration of pancreatic tissue.

However, the question remains what is the mechanism by which ghrelin prevents entering of pancreatic enzymes to the circulation. In physiological condition, zymogen granules containing inactive pancreatic enzymes are secreted by exocytosis at the apical part of pancreatic acinar cells to the lumen of pancreatic acini. In the case of acute pancreatitis this process is impaired. Partly condensed vacuoles with newly synthesized digestive enzymes accumulate at the basolateral part of the acinar cell and form the large vacuoles containing digestive enzymes and lysosomal hydrolase, cathepsin D [54]. Later, mature zymogen granules and lysosomes also fuse with these large cathepsin D-containing vacuoles [55,56,57]. Lysosomal enzymes lead to premature intracellular activation of pancreatic enzymes and cellular organelles damage. Subsequently, these large vacuoles and individual zymogen granules fuse with the basolateral plasma membrane, discharging their content into the interstitial space and developing acute pancreatitis [54,57,58]. In the next step, active pancreatic digestive enzymes enter the bloodstream. These findings are in harmony with our current studies. In agreement with previous studies, we observed that administration of cerulein led to vacuolization of acinar cells. Vacuolization of acinar cells should be recognized as an index of colocalization of lysosomes and zymogens in the large vacuoles and intracellular activation of pancreatic enzymes. Our present study also showed that pretreatment with ghrelin before cerulein administration reduces vacuolization of acinar cells. This finding is an evidence that ghrelin decreases the cerulein-induced premature activation of pancreatic enzymes within acinar cells.

Our current studies also showed that administration of ghrelin reduces inflammatory leukocyte infiltration of the pancreas in cerulein-induced pancreatitis. Reduction in inflammatory infiltration decreases pancreatic damage and for this reason also reduces premature intrapancreatic activation of digestive enzymes and release of these enzymes into the circulation. Moreover, the development of acute pancreatitis is associated with generation of reactive oxygen species [59]. Excess free oxygen radicals cause oxidative damage of cellular lipids, proteins and nucleic acids. Peroxidation of cellular lipids is the first step of reactive oxygen species-mediated cellular damage. Previous studies indicated that treatment with ghrelin reduces lipid peroxidation and increases the activity of enzymes involved in deactivation of free oxygen radicals [29]. These findings show a further mechanism of action of ghrelin, which leads to a decrease in plasma level of active pancreatic enzymes.

Tumor necrosis factor-α (TNF-α) is the best-known member of a cytokine family called the tumor necrosis factor superfamily [60]. TNF-α is a cell signaling cytokine involved in wide spectrum of responses to stress and injury. It is mainly produced by macrophages and other immune cells, such as monocytes, lymphocytes, natural killer cells, neutrophils and eosinophils. However, TNF-α can be also produced by many other cell types, including pancreatic acinar cells [61]. This cytokine exhibits host-damaging effects in different autoimmune and inflammatory diseases. TNF-α is an activator of immune cells and regulates the synthesis of other pro-inflammatory cytokines and leukocyte adhesion molecules. It is an endogenous pyrogen and, for this reason, it may induce fever. Moreover, this pro-inflammatory cytokine activates the cell death signaling, leading to cell apoptosis. It may induce cachexia and inhibit tumorigenesis, and viral replication [60,62]. Numerous studies indicate that TNF-α plays a pivotal role in the development of severe acute pancreatitis. Measurement of serum TNF-α concentration was suggested to be useful biomarker in predicting severe pancreatitis and development of multiorgan failure or septic shock [63]. However, TNF-α is rapidly cleared from the circulation and for this reason the potential prognostic value of its measurement is limited to the first days after the onset of the disease [62,63].

In our current study, we found that pretreatment with ghrelin in rats with intact sensory nerves reduces the pancreatitis-induced increase in plasma concentration of TNF-α. This finding was in line with our observation that administration of ghrelin reduces pancreatic leukocyte infiltration. In addition, these findings taken together indicate that protective effect of ghrelin in cerulein-induced pancreatitis in sensory nerves-intact rats involves anti-inflammatory effect of this polypeptide.

The next interesting finding of our current study is observation that induction of acute pancreatitis by cerulein increases plasma level of interleukin-4 (IL-4) and pretreatment with ghrelin dose-dependently enhances this effect. On the other hand, ghrelin did not affect plasma level of IL-4 in rats without induction of acute pancreatitis. Previous studies showed that IL-4 exhibits anti-inflammatory effects. Hart et al. reported that IL-4 suppresses the production of pro-inflammatory interleukin-1β (IL-1β) and TNF-α by monocytes stimulated with lipopolysaccharide and interferon γ, and this effect is similar to that obtained with dexamethasone [64]. Similar effects were obtained by Vannier et al. [65]. They found that IL-4 inhibits the lipopolysaccharide-induced synthesis of IL-1β in human peripheral blood mononuclear cells and enhanced the lipopolysaccharide-induced synthesis of anti-inflammatory IL-1 receptor antagonist. Anti-inflammatory effect of IL-4 was also found in acute pancreatitis. Protective and therapeutic effect of IL-4 in severe taurocholate-induced acute pancreatitis was reported by Zhang et al. [66]. IL-4 reduced necrosis in this model of acute pancreatitis and authors suggested that this effect was related to enhanced expression of complement regulatory proteins, CD55 and CD59. In addition, clinical studies indicate that IL-4 affects the development and course of acute pancreatitis. Acute pancreatitis, as well as endoscopic retrograde cholangiopancreatography (ERCP) increases serum level of interleukin-4. Kilciler et al. found that 24 h after ERCP, a level of interleukin-4 is significantly lower in patients with post-ERCP pancreatitis than in those without pancreatitis [67]. This observation suggests that the development of acute pancreatitis is the effect of imbalance between pro- and anti-inflammatory factors, while maintaining a balance between those factors prevent the development of inflammation. Too low level of anti-inflammatory IL-4 or too low increase in serum level of this cytokine in response to the presence of pro-inflammatory factors can disturb this balance and lead to the development of acute pancreatitis and/or increase in its severity. On the other hand, IL-4 was shown to stimulate the class of macrophages named alternatively activated macrophages [68]. Alternatively activated macrophages are involved in dampening inflammation and promotion of wound healing, fibrosis, and tumorigenesis. Study of Xue et al. [68] showed that mouse and human pancreatic stellate cells are source of IL-4/IL-13 and a cross talk between macrophages and pancreatic stellate cells is associated with the development and progression of chronic pancreatitis. Our present study showed that ghrelin enhances the pancreatitis-evoked increase in plasma level of IL-4. Those data could suggest that administration of ghrelin may promote the development of chronic pancreatitis. Fortunately, our present study also showed that ghrelin does not affect plasma level of interleukin-4 in rats without induction of acute pancreatitis. For this reason, the probability that administration of ghrelin may stimulate the development of chronic pancreatitis is minimal.

Numerous clinical [69,70,71,72] and experimental [73,74,75,76] studies showed that disturbance in pancreatic microcirculation can be a primary cause of acute pancreatitis. Moreover, pancreatic microcirculation disorder and pancreatic ischemia are observed in acute pancreatitis evoked by primary non-vascular mechanism, and always these alterations increase the severity of this disease [73,74,77]. On the other hand, the improvement of blood flow through pancreatic vessels inhibits the development of acute pancreatitis and accelerates the recovery in this disease [43,78,79]. Our present study showed that after a 5-h administration of cerulein, pancreatic blood flow was reduced by around 40% in comparison to value observed in sensory nerves-intact rats without induction of acute pancreatitis. Pretreatment with ghrelin given intraperitonelly at the dose of 12.5, 25 or 50 µg/kg was without any significant effect on pancreatic blood flow in sensory nerves-intact rats without induction of acute pancreatitis, as well as in rats with induction of acute pancreatitis. We only found that pretreatment with ghrelin exhibits an insignificant tendency to improve pancreatic blood flow in sensory nerves-intact rats with acute pancreatitis. These observations are in agreement with previous studies showing lack of effect of pretreatment with ghrelin on pancreatic blood flow in cerulein- or ischemia/reperfusion-induced pancreatitis [24,26]. In contrast, long-term treatment with ghrelin after induction of cerulein- or ischemia/reperfusion-induced pancreatitis improved pancreatic blood flow [27,28]. These data taken together with our current observation suggests that influence of ghrelin on pancreatic blood flow in the course of acute pancreatitis is indirect effect likely due to improvement of pancreatic morphology. For this reason, short term observation was not able to demonstrate any effect in pancreatic circulation after a single dose of ghrelin.

We may also speculate that the lack of beneficial effects of ghrelin on pancreatic blood flow is associated with activation of coagulation in pancreatic vessels. Numerous experimental and clinical studies showed that coagulative disorders occur in acute pancreatitis [80,81,82,83,84]. Acute pancreatitis stimulates intravascular coagulation with formation of thrombi within blood vessels and coagulation disorders may be in the range between scattered intravascular thrombosis in pancreatic microcirculation to severe disseminated intravascular coagulation [84]. Moreover, animals and clinical studies showed that treatment with heparin or anticoagulants inhibits the development of acute pancreatitis and accelerates the recovery in this disease [85,86,87,88,89,90,91,92,93]. These findings indicate that disorders of coagulation play the important role in acute pancreatitis and these disorders may be responsible for the lack of significant improvement in pancreatic blood flow in animals treated with ghrelin despite improved histology of the pancreas.

The major finding of current research was to determine the role of sensory nerves in the protective effect of ghrelin in cerulein-induced pancreatitis. Ablation of sensory nerves by neurotoxic doses of capsaicin performed before induction of acute pancreatitis significantly reduced plasma concentration of anti-inflammatory IL-4 and showed a tendency to an additional increase in pancreatic weight and decrease in pancreatic blood flow. However, the last two changes were not statistically significant. Other parameters tested were not affected by ablation of sensory nerves and those observation are similar to that observed previously in cerulein-induced pancreatitis [40,42,43]. These data indicate that ablation of sensory nerves reduces endogenous anti-inflammatory mechanisms in the pancreas and moderately increases pancreatic damage in acute pancreatitis. On the other hand, ablation of sensory nerves totally abolished the protective effect of ghrelin in this model of acute pancreatitis. In rats with ablation of sensory nerves before induction of acute pancreatitis, pretreatment with ghrelin was without any effect on morphological signs of pancreatic damage, pancreatic weight, plasma level of pancreatic digestive enzyme, lipase or plasma concentration of TNF-α and IL-4. These observations indicate that sensory nerves mediate protective effect of ghrelin in the pancreas. This concept is supported by study performed by Date et al. [94]. They found the presence of ghrelin receptors in vagal afferent neurons. Vagotomy or capsaicin-induced deactivation of the gastric vagal afferent neurons eliminated ghrelin-induced food intake, growth hormone secretion, and activation of neuropeptide Y- and GH-RH-producing neurons [94].

Our current studies showed that ablation of sensory nerves by neurotoxic doses reduces pancreatic blood flow in basal condition without induction of acute pancreatitis. In rats treated with ghrelin after deactivation of sensory nerves, pancreatic blood flow was significantly reduced in comparison to a level observed in sensory nerves-intact rats without induction o acute pancreatitis and treated with saline or ghrelin. This observation brought two important pieces of information First, it indicates that, under basic conditions, sensory nerves exhibit tonic activity and participate in maintaining adequate blood flow in the pancreas. This effect seems to be related to the ability of the sensory nerves to local release of vasoactive neuromediators, mainly calcitonin gene-related peptide (CGRP) [31,32,33]. Stimulation of sensory nerves results in the release of these neuromediators, leading to vasodilatation and increase in organ blood flow [40]. Second, ablation of sensory nerves decreases plasma and tissue level of CGRP [35]. Results of this part of the study also showed that administration of ghrelin is not able to reverse the negative effect of sensory nerve ablation on pancreatic blood flow. This lack of improvement in blood flow following administration of ghrelin additionally supports the thesis about the essential role of sensory nerves in the protective action of ghrelin in the pancreas.

The last part of our present study was conducted to determine the effect of sensory nerve ablation and the administration of ghrelin, performed in rats before isolation of pancreatic acini, on acinar cell expression of mRNA for TNF-α and heat shock protein 70 (HSP70) and synthesis of those proteins. Exposure of isolated pancreatic acini to cerulein led to increase in cellular expression of mRNA for TNF-α, as well as cellular synthesis of TNF-α. In acinar cells obtained from sensory nerves-intact rats pretreated with ghrelin, expression mRNA for TNF-α and synthesis of this pro-inflammatory cytokine after incubation with cerulein was reduced. On the other hand, pretreatment with ghrelin was without any effect on expression of mRNA for TNF-α and synthesis of TNF-α under basic condition without the presence of cerulein. Ablation of sensory nerves, before isolation of pancreatic acini, abolished the inhibitory effect of ghrelin on the cerulein-induced expression of mRNA for TNF-α and synthesis of this protein.

These observations provide some important information. First, these data indicate that acinar cells may play the active role in the development of inflammation in acute pancreatitis by releasing pro-inflammatory cytokine, TNF-α. This finding is agreement with observations of Gukovskaya et al. [95]. They found that pancreatic acinar cells produce, release, and respond to TNF-α. In addition, this part of our study brings additional evidence that ghrelin exhibits the anti-inflammatory and protective effect in acute pancreatitis. Our present study also showed that protective effect of ghrelin on the pancreas involves the action of ghrelin on the level of pancreatic acinar cells. Moreover, these in vitro studies confirm and extend our current observations obtained in the first part of our research in animal model of acute pancreatitis that the protective effect of ghrelin in acute pancreatitis is mediated by capsaicin-sensitive sensory nerves.

The next interesting discovery of our current study is the observations on the effect of ghrelin on expression and production of HSP70 in isolated pancreatic acini. HSPs are highly conserved cytoprotective proteins. Their synthesis can be induced by various stressors such as hyper- and hypothermia, toxins, heavy metals, and free radicals [96]. HSPs play a role of molecular chaperons in folding newly synthesized cell proteins and assisting in the refolding of damaged proteins. HSP70 is located in all cellular compartments, including the endoplasmic reticulum, mitochondria, cytosol and nucleus [97]. Previous studies performed on the animals demonstrated that both, thermal and non-thermal stresses inhibit the development of cerulein-induced pancreatitis and prevent premature trypsinogen activation in pancreatic tissue and this effect was shown to be mediated by HSP70 [98]. HSPs are also involved in thermal-induced protection against arginine- and taurocholate-induced acute pancreatitis [99,100]. Protective effect of ischemic preconditioning in the pancreas is associated with increase in synthesis of HSP70 [101]. The protective mechanisms of HSPs in the pancreas include stabilization and refolding of damaged proteins, resistance of cells to apoptosis or necrosis, decrease in the level of pro-inflammatory cytokines, antioxidant effects and inhibition of zymogen activation within pancreatic acinar cells [96,98,102]. Our present observation that incubation of isolated pancreatic acini with cerulein induces production of HSP70 in acinar cells is in harmony with previous studies showing the increase in HSP70 at the mRNA and protein level in the pancreas in cerulein-induced acute pancreatitis [103,104]. The major finding of this part of our present research is observation that administration of ghrelin stimulates the expression of mRNA for HSP70 and production of HSP70 in pancreatic acini isolated from rats with intact sensory nerves. This effect was found in acini incubated in a cerulein-depleted medium. Moreover, the administration of ghrelin augmented the stimulatory effect of cerulein on HSP expression and production. In contrast, ghrelin failed to induce expression and production of HSP70 in pancreatic acini obtained from rats with ablated capsaicin-sensitive sensory nerves. These findings are the next evidence that the maintenance of sensory nerves integrity is necessary for protective effect of ghrelin in the pancreas.

## 4. Material and Methods

The research was conducted in accordance with the protocols approved by the Jagiellonian University Ethical Committee for Research and Animal Ethics. Experiments were carried out in two series: in vivo and in vitro studies.

### 4.1. In Vivo Studies

#### 4.1.1. Animals and Treatment

In vivo studies were performed on male Wistar rats weighing 170–200 g. Rats were housed in cages in standard conditions at room temperature with a normal circadian rhythm; 12-h day/night cycle. During a one-week period of acclimation to a new environment, food (commercial pellet chow) and water were available ad libitum.

All experimental procedures performed in this study were approved by the Jagiellonian University Ethical Committee on Animals Experimentation (Permit No ZI/UJ/118/2001 released on July 20, 2001). Animals were deprived of food with free access to water 24 h prior to the start of experiment.

Rats participating in in vivo studies were randomly divided into 11 experimental groups (each containing 10–15 animals) as follows:

(1) Control group: sensory nerves-intact rats treated with saline given as a single intaperitoneal (i.p.) injection followed by subcutaneous (s.c.) infusion for 5 h;
(2) Sensory nerves-intact rats treated with ghrelin given at a dose of 12.5 µg/kg as single i.p. injection followed by s.c. infusion of saline for 5 h;
(3) Sensory nerves-intact rats treated with ghrelin given at a dose of 25 µg/kg as single i.p. injection followed by s.c. infusion of saline for 5 h;
(4) Sensory nerves-intact rats treated with ghrelin given at a dose of 50 µg/kg as single i.p. injection followed by s.c. infusion of saline for 5 h;
(5) Sensory nerves-intact rats treated with saline given as single i.p. injection followed by induction of acute pancreatitis (AP) by s.c. infusion of cerulein for 5 h;
(6) Sensory nerves-intact rats treated with ghrelin given at a dose of 12.5 µg/kg as single i.p. injection followed by induction of AP by s.c. infusion of cerulein for 5 h;
(7) Sensory nerves-intact rats treated with ghrelin given at a dose of 25 µg/kg as single i.p. injection followed by induction of AP by s.c. infusion of cerulein for 5 h;
(8) Sensory nerves-intact rats treated with ghrelin given at a dose of 50 µg/kg as single i.p. injection followed by induction of AP by s.c. infusion of cerulein for 5 h;
(9) Rats with capsaicin deactivation of sensory nerves (CDSN) treated with saline given as single i.p. injection followed by induction of AP by s.c. infusion of cerulein for 5 h;
(10) Rats with CDSN treated with ghrelin given at a dose of 50 µg/kg as single i.p. injection followed by induction of AP by s.c. infusion of cerulein for 5 h; and
(11) Rats with CDSN treated with ghrelin given at a dose of 50 µg/kg as single i.p. injection followed by s.c. infusion of saline for 5 h.

Acute pancreatitis (AP) was induced by s.c. infusion of cerulein (Takus, Pharmacia, GmbH, Erlangen, Germany) at a dose of 5 µg/kg/h for 5 h. Cerulein was diluted in saline and infused at a rate of 1 mL/h. Control groups received s.c. infusion of saline for 5 h (1 mL/h) as described previously [105]. Thirty minutes before the start of cerulein or saline infusion, rats were treated with tramadol (Poltram, Polpharma, Starogard Gdański, Poland) given intramuscularly at a dose of 1 mg/kg to minimize pain and distress. During s.c. infusion of cerulein or saline, conscious rats were placed in individual Bollman cages. All experiments were carried out in the morning.

Capsaicin deactivation of sensory nerves (CDSN) was performed by neurotoxic doses of capsaicin (Fluka, Buchs, Switzerland) given s.c. during 3 days at a total dose of 100.0 mg/kg, 7 days prior to induction of acute pancreatitis or infusion of saline [106].

Ghrelin (Bachem AG, Budendorf, Switzerland) dissolved in 0.5 mL of 0.9% NaCl, was given i.p. to rats with intact sensory nerves or rats with CDSN, 30 min before the infusion of 0.9% NaCl (control group) or cerulein (AP). Rats with intact sensory nerves were treated with ghrelin given at a dose of 12.5, 25 or 50 µg/kg. Rats with CDSN were treated with ghrelin given at one dose of 50 µg/kg, because this dose exhibited the greatest protective effect on the pancreas in rats with intact sensory nerves.

#### 4.1.2. Determination of Pancreatic Blood Flow

Following 5-h infusion of saline or cerulein, the rats were anesthetized with pentobarbital (30 mg/kg i.p., Vetbutal, Biowet, Puławy, Poland) and then the abdominal cavity was opened. Pancreatic blood flow was measured by a laser Doppler flowmeter using a Laserflo, model BPM 403 A (Blood Perfusion Monitor, Vasdamedics Inc., St. Paul, MN, USA) as previously described [107]. Blood flow was measured in five different pancreatic regions in each rat and expressed as the percent change of the control value.

#### 4.1.3. Biochemical Parameters

Immediately after the measurement of pancreatic blood flow, blood samples were taken from the inferior vena cava for plasma measurement of lipase, tumor necrosis factor-α (TNF-α) and interleukin-4 (IL-4).

Lipase activity was determined using LIPA DT Slides (Vitros DT Chemistry System, Johnson & Johnson Clinical Diagnostic, Inc., Rochester, NY, USA). The reading was made using the Kodak Ectachem DT II System analyzer (Eastman Kodak Company, Rochester, NY, USA) [108].

TNF-α and IL-4 concentrations were determined by immunoenzymatic assay (ELISA) using rat diagnostic tests (Biosource International, Camarillo, CA, USA) [107].

#### 4.1.4. Pancreatic Weight and Histological Examination

The pancreas was carefully isolated from the abdominal cavity of each animal, purified from adipose tissue, rinsed in 0.9% NaCl, filtered and weighed.

Histological studies were carried out on pancreatic samples fixed in 10% formalin and stained with hematoxylin and eosin (H&E). Specimens were examined by a professional pathologist. Histological grading of pancreatic edema, leukocyte inflammatory infiltration, vacuolization of acinar cells was made using a scale ranging from 0 to 3 as described previously in detail [108,109]:

1. pancreatic edema (0—no edema, 1—interlobar edema, 2—interlobar and moderate intralobular edema, 3—severe interlobula and intralobular edema);
2. leukocyte inflammatory infiltration (0—Absent, 1—scarce perivascular infiltration, 2—moderate perivascular and scarce diffuse infiltration, 3—abundant diffuse infiltration);
3. vacuolozation of acinar cells (0—absent, 1—involving less than 25% of acinar cells, 2—involving from 25 to 50% acinar cells, 3—involving more than 50% of acinar cells);
4. necrosis of acinar cells (0—absent, 1—involving less than 15% of acinar cells, 2—involving from 15 to 35% acinar cells, 3—involving more than 35% of acinar cells); and
5. hemorrhage (0—absent, 1 from 1 to 2 foci per slide, 2—from 3 to 5 foci per slide, 3—more than 5 foci per slide).

The results of histological examination were shown as predominant histological grading (mode) of pancreatic edema, inflammatory infiltration and vacuolization of acinar cells in each experimental group (Table 1). Moreover, we showed the representative morphological features of the pancreas in main experimental groups in Figure 2 and the total histological score of pancreatic damage calculated as the sum of degrees of pancreatic edema, inflammatory infiltration and vacuolization of acinar cells as described previously [110].

### 4.2. In Vitro Studies

Isolated acinar cells were obtained from rats with intact sensory nerves or rats with CDSN. Rats were treated with ghrelin given i.p. at single dose of 50 µg/kg or with saline and 48 h later as described previously [111], animals were again anesthetized with pentobarbital. The abdominal cavity was opened and the pancreas was carefully dissected out from its attachment to the stomach, duodenum and spleen. Pancreatic acinar cells were isolated by collagenase digestion as described previously [112,113] and incubated in cerulein-free or cerulein containing solution. Previous studies showed that cerulein given at a concentration of 10^−8^ M and 5-h incubation were most effective in stimulating exocrine secretion and gene expression in pancreatic acinar cells [114,115], thus these concentration of cerulein and time of incubation were selected for our current in vitro studies. Studies in each experimental group were repeated at least six times.

During the in vitro studies, we used following experimental groups:

(1) acinar cells obtained from control sensory nerves-intact rats treated i.p. with saline; after isolation, acinar cells incubated in cerulein-free solution;
(2) acinar cells obtained from sensory nerves-intact rats treated i.p. with ghrelin at a dose of 50 µg/kg; after isolation, acinar cells incubated in cerulein-free solution;
(3) acinar cells obtained from sensory nerves-intact rats treated i.p. with saline; after isolation, acinar cells incubated in solution containing cerulein at a concentration of 10^−8^ M;
(4) acinar cells obtained from sensory nerves-intact rats treated i.p. with ghrelin at a dose of 50 µg/kg; after isolation, acinar cells incubated in solution containing cerulein at a concentration of 10^−8^ M;
(5) acinar cells obtained from rats with CDSN treated i.p. with saline; after isolation, acinar cells incubated in solution containing cerulein at a concentration of 10^−8^ M;
(6) acinar cells obtained from rats with CDSN treated i.p. with ghrelin at a dose of 50 µg/kg; after isolation, acinar cells incubated in solution containing cerulein at a concentration of 10^−8^ M; and
(7) acinar cells obtained from rats with CDSN treated i.p. with ghrelin at a dose of 50 µg/kg; after isolation, acinar cells incubated in cerulein-free solution.

#### 4.2.1. Determination of *TNF-α* and *Heat Shock Protein 70 (HSP70)* Gene Expression by Reverse Transcription-Polymerase Chain Reaction (RT-PCR)

The isolation of total cellular RNA was performed employing TRIzol Reagent (Gibco-BRL, Life Technologies, Gaithersburg, MD, USA) in accordance with the manufacturer’s protocol [116]. Precipitated in the final step of isolation, RNA was resuspended in RNase-free water. Final concentration of the RNA isolates was estimated by the measurement of absorbance at 260 nm wavelength. To establish the purity of the isolates calculation of A260/A280 ratio was done. The integrity of RNA isolates was confirmed by electrophoresis in formaldehyde denaturizing conditions.

cDNA synthesis was performed employing the Reverse Transcription System (Promega Corp., Madison, WI, USA) using 1 µg of RNA according to the manufacturers protocol. Two of the cDNAs were used for polymerase chain reaction. Promega PCR reagents were used in all PCR reactions. Specific primers were synthesized by Sigma-Genosys (Pampisford, UK). Semi-quantitative analysis of the abundance of cDNA product for each sample was performed using Foto/Analyst Fotodyne System (Fotodyne Inc., Hartland, WI, USA) on ethidium bromide stained 2% agarose gel. O’Gene Ruler 50 bp DNA Ladder (Fermentas GmbH, St. Leon-Rot, Germany) was used to confirm the predicted location of the PCR products. Results of the semi-quantitative analysis were expressed as a ratio using *β-actin* gene product as a reference for each sample. PCR negative and positive reaction controls were added in all experiments to confirm specificity of reaction.

The gene, primer sequences, product length, characteristic annealing temperatures and references are summarized in Table 2.

#### 4.2.2. Determination of TNF-α and HSP70 Protein Synthesis by Immunoblotting

The whole cell protein extracts from rat pancreatic acinar cells were prepared as described previously [119]. Samples containing 5–10 μg of proteins were separated under denaturing condition in 12% polyacrylamide gel. Following separation samples were transferred onto the PVDF membrane (BioRad, Hecules, CA, USA) and membranes were blocked for 2 h at room temperature in blocking buffer (5% non-fat dried milk in PBS). Membranes containing immobilized protein samples were exposed to the primary antibody diluted 1:1000 for 1 h at room temperature on the agitating platform. After antibody probing membranes were washed three times for 10 min in TBST buffer (0.1 M Tris pH 8.0; 1.5 M NaCl; 0.5% TritonX-100). Suitable secondary antibody in the dilution 1:5000 in blocking buffer was applied for 1 h at room temperature. Following the secondary antibodies probing, washing procedure was performed as described above. Each blot was stripped and probed with GAPDH antibody to confirm equal protein loading. Proteins complexed with antibodies were detected using Super Signal West Pico Chemiluminescent Substrate Thermo Fisher Scientific (Waltham, MA, USA) according to the manufactures protocol. All presented results were obtained in six consecutive experiments.

All primary antibodies: mouse monoclonal IgG1 anti GAPDH (A-3); goat polyclonal IgG anti TNF–α (R–19); mouse monoclonal IgG1 anti HSP70 (3A3) as well as secondary goat anti-mouse IgG1-HRP conjugated and rabbit anti-goat IgG-HRP conjugated were purchased from Santa Cruz Biotechnology (Santa Cruz, CA, USA).

### 4.3. Statistical Analysis

Results are expressed as means ± SEM. A statistical analysis was done by one-way analysis of variance (ANOVA), followed by Tukey’s multiple comparison test. A statistical analysis was conducted using the statistical package GraphPad Prism (GraphPad Software, San Diego, CA, USA). Differences with *p* < 0.05 were considered significant.

## 5. Conclusions

Our present study has demonstrated that ghrelin exhibits the protective effect in cerulein-induced acute pancreatitis on the organ and pancreatic acinar cell level. The maintenance of sensory nerve integrity is necessary for this effect.

## Figures and Tables

**Table 1:** Effect of saline or ghrelin (GHRL) given intraperitoneally at increasing doses of 12.5, 25 or 50 µg/kg on morphological changes of pancreatic tissues in the course of cerulein-induced pancreatitis (CIP) in animals with intact sensory nerves or with capsaicin deactivation of sensory nerves.

Groups	Edema (0–3)	Infiltration (0–3)	Vacuolization (0–3)
Intact Sensory Nerves			
Control (saline)	0	0	0
CIP	2–3	2–3	3
GHRL 12.5 µg/kg	0	0	0
GHRL 25 µg/kg	0	0	0
GHRL 50 µg/kg	0	0	0
GHRL 12.5 µg/kg + CIP	2–3	2	2–3
GHRL 25 µg/kg + CIP	2	1–2	2–3
GHRL 50 µg/kg + CIP	1–2	1–2	2
Capsaicin Deactivation of Sensory Nerves			
Saline	0	0	0
CIP	2–3	2–3	3
GHRL 50 µg/kg + CIP	3	2–3	3
GHRL 50 µg/kg	0	0	0

Numbers represent the predominant histological grading in each experimental group.

**Table 2:** Gene, primers’ sequences, product length, annealing temperatures, references.

Gene	Sequence 5′ → 3′	Product (bp)	Annealing Temperature (°C)	Reference
*β-actin*	Sense: TTG TAA CCA ACT GGG ACG ATA TGG	764	60	[117]
	Antisense: GAT CTT GAT CTT CAT GGT GCT AGG			
*TNF-α*	Sense: TAC TGA ACT TCG GGG TGA TTG GTC C	295	60	[118]
	Antisense: CAG CCT TGT CCC TTG AAG AGA ACC			
*HSP70*	Sense: GTG AAG ATC TGC GTC TGC TTG	590	60	[104]
	Antisense: TTT GAC AAC AGG CTG GTG AAC C			
