# Correction: Bonior, J., et al. Capsaicin-Sensitive Sensory Nerves Are Necessary for the Protective Effect of Ghrelin in Cerulein-Induced Acute Pancreatitis in Rats. *Int. J. Mol. Sci.* 2017, *18*, 1402

**DOI:** 10.3390/ijms20123089

**Published:** 2019-06-25

**Authors:** Joanna Bonior, Zygmunt Warzecha, Piotr Ceranowicz, Ryszard Gajdosz, Piotr Pierzchalski, Michalina Kot, Anna Leja-Szpak, Katarzyna Nawrot-Porąbka, Paweł Link-Lenczowski, Michał Pędziwiatr, Rafał Olszanecki, Krzysztof Bartuś, Rafał Trąbka, Beata Kuśnierz-Cabala, Artur Dembiński, Jolanta Jaworek

**Affiliations:** 1 Department of Medical Physiology, Faculty of Health Sciences, Jagiellonian University Medical College, 12 Michałowskiego St., 31-126 Krakow, Poland; joanna.bonior@uj.edu.pl (J.B.); piotr.pierzchalski@uj.edu.pl (P.P.); m.kot@uj.edu.pl (M.K.); a.leja-szpak@uj.edu.pl (A.L.-S.); k.nawrot-porabka@uj.edu.pl (K.N.-P.); p.link-lenczowski@uj.edu.pl (P.L.-L.); jolanta.jaworek@uj.edu.pl (J.J.); 2 Department of Physiology, Faculty of Medicine, Jagiellonian University Medical College, 16 Grzegórzecka St., 31-531 Krakow, Poland; mpwarzec@cyf-kr.edu.pl (Z.W.); mpdembin@cyf-kr.edu.pl (A.D.); 3 Department of Emergency Medical Care, Faculty of Health Sciences, Jagiellonian University Medical College, 12 Michałowskiego St., 31-126 Krakow, Poland; ryszard.gajdosz@uj.edu.pl; 4 2nd Department of Surgery, Faculty of Medicine, Jagiellonian University Medical College, 21 Kopernika St., 31-501 Krakow, Poland; michal.pedziwiatr@uj.edu.pl; 5 Department of Pharmacology, Faculty of Medicine, Jagiellonian University Medical College, 16 Grzegórzecka St., 31-531 Krakow, Poland; rafal.olszanecki@uj.edu.pl; 6 Department of Cardiovascular Surgery and Transplantology, Faculty of Medicine, Jagiellonian University, JP II Hospital, 80 Prądnicka St., 31-202 Krakow, Poland; krzysztof.bartus@uj.edu.pl; 7 Department of Rehabilitation, Faculty of Health Sciences, Jagiellonian University Medical College, 3 Koło Strzelnicy St., 30-219 Krakow, Poland; rafal.trabka@uj.edu.pl; 8 Department of Diagnostics, Chair of Clinical Biochemistry, Faculty of Medicine, Jagiellonian University Medical College, 15 A Kopernika St., 31-501 Krakow, Poland; mbkusnie@cyf-kr.edu.pl

We would like to submit the correction to our published paper [1]. The reason for the correction is an error in the histological images presented in this article. Two histological images (old Figure 2A,D) are incorrect and for this reason they should be replaced with the correct new figure (Figure 1).

The above errors were without material impact on the final results and conclusions of our papers. We apologize for this inconvenient situation. 

## Figures and Tables

**Figure 1:**
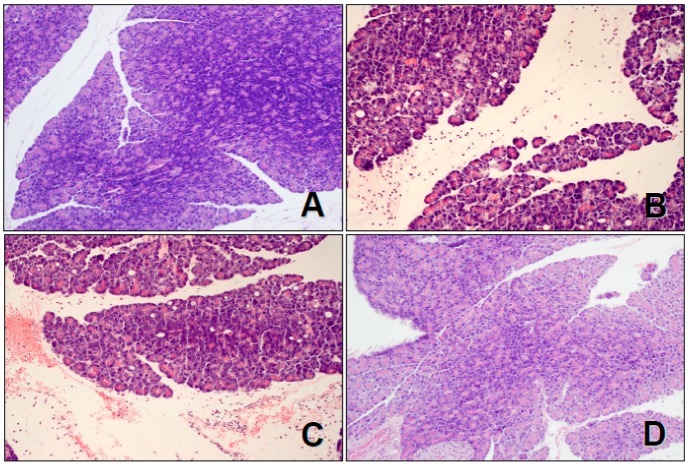
Histological images of pancreatic tissues stained with hematoxylin and eosin, magnification 400×: (**A**) control sensory-nerves-intact rats treated with saline without cerulein-induced pancreatitis (CIP); (**B**) sensory-nerves-intact rats treated with saline followed by CIP development; (**C**) rats with capsaicin deactivation of sensory nerves treated with saline followed by CIP development; and (**D**) sensory-nerves-intact rats treated with ghrelin given at a dose of 50 µg/kg followed by CIP development.
